# Mass treatment for trachoma: how does it all work?

**Published:** 2013

**Authors:** Hillary K Rono

**Affiliations:** Ophthalmologist, Kitale District Hospital, Kitale, Kenya.

**Figure:**
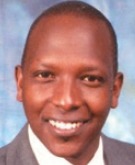
Hillary K Rono

Trachoma is the leading infectious cause of blindness and is endemic in 53 countries. An estimated 325 million people live in areas where they can be exposed to trachoma, and more than 7 million suffer from trichiasis, the final painful stage of this eye disease.

The World Health Organization (WHO) initiated a global programme to eliminate trachoma by 2020. At its core is the SAFE strategy: Surgery, Antibiotics, Facial cleanliness and Environmental improvement to reduce transmission. Trachoma control efforts have increased with mass drug administration (MDA) of azithromycin (Zithromax), an antibiotic donated by Pfizer Inc. The goal is 80% coverage in endemic areas for at least 3 years.

To maximise coverage, programme managers and health workers must understand the community's knowledge and health priorities as well as their attitudes and beliefs.

This has been well demonstrated in other successful public health programmes in Africa, e.g. the Africa Program for Onchocerciasis Control (APOC)^1^ and the Guinea Worm Program (GWP).^2^

This article distils what has been learned from MDA programmes in Kenya, where trachoma control activities have been initiated in eight districts.

Successful MDA programmes depend on completing all of the following activities in each district where MDA will take place.

## 1. Planning

Health workers in managerial positions, also referred to as the District Health Management Team (DHMT), gather to do the following:

- choose drug distribution points and drug storage sites
- determine the target population for each location and distribution point
- procure the drugs (azithromycin)
- determine the human resources needed
- select divisional coordinators and supervisors
- prepare the distribution budget
- meet with the partner(s) supporting MDA, which may be an NGO and/or a district or regional authority.

## 2. Sensitisation of stakeholders and mobilisation of the community

The DHMTs are responsible for creating awareness about MDA. This is aimed at everyone involved in – and affected by-the MDA programme.

In each division (a population of around 50,000 people) the DHMT organises workshops with local chiefs and assistant chiefs and ask them to encourage their communities to get involved. The staff member designated as the divisional coordinator explains the purpose of the MDA, the reasons for choosing this area, and the drugs being used. They also discuss the potential side effects of the drugs to dispel negative information and perceptions.

We have found that it is best to engage everyone in discussion, rather than merely giving a lecture. This gives community members a chance to express any fears or concerns they may have.

**Figure:**
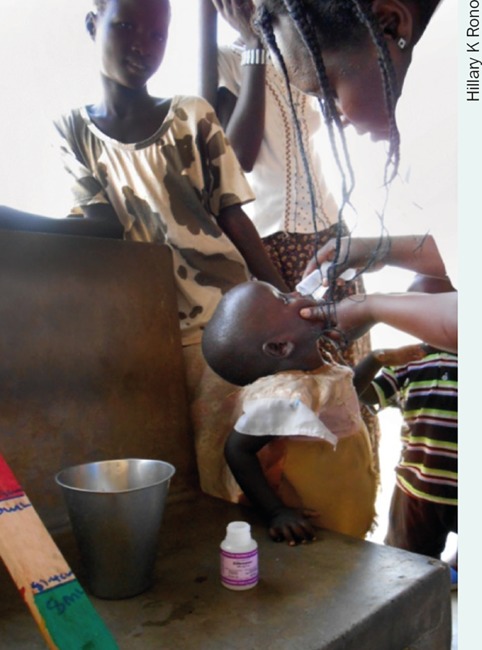
A community health extension worker (CHEW) in Kataboi dispensary, Turkana County, gives azithromycin to a child during mass drug administration. KENYA

If there are several ethnic groups in the target area these may need different approaches in order that they understand and participate in distribution. Protecting the future generation from disease is a message that resonates well in most communities.

Speaking to faith-based organisations about MDA, and engaging the local media and opinion leaders, are also important in getting a community ready to actively support distribution and to accept the drugs. This is also known as community mobilisation. Community Health Extension Workers (CHEWs) in the area will assist by educating the community about the importance of hygiene and sanitation.

## 3. Recruitment of volunteers and community mapping

First, the DHMT members involved in managing the MDA meet to discuss the number of CHEWs and community volunteers needed (Table 1) and what training they might require. Chiefs, assistant chiefs, and local opinion leaders help to choose the community volunteers. In communities where there are already trained community health workers, the community volunteers will be chosen from among them.

Community mapping involves assessing the population in an area and identifying public facilities such as schools, clinics and churches that can be used as distribution points. Distribution dates are determined, based on places or activities that bring people together, such as market days.

**Table 1.** Personnel involved in MDA at district level in Kenya

**District Health Management Team (DHMT).** The team of health managers and leaders who oversee mass drug administration in a **district** (population of 300,000 to 600,000). Divisional coordinators and some supervisors are also members.
**Divisional coordinator.** Responsible for training of community volunteers and CHEWs and for community mobilisation in the **division** (population of around 50,000 people). Oversees the activities of 8-10 supervisors during MDA.
**Supervisor.** Oversees 6-10 distribution teams in one **location** (population of around 10,000 people). Liaises with chiefs and assistant chiefs and helps to ensure logistical support is available to teams. Reports to divisional coordinator.
**Community Health Extension Workers (CHEWs).** They are trained nurses and/or public health officers or technicians who work at the health facilities (dispensaries and health centers) in the community. They are employed by the health system and are the primary contact between the community and health system.
**Community volunteers.** They are community members, chosen by their community, who offer their services as volunteers. They are concerned with the welfare of the people in relation to improving health and preventing illness.

## 4. Training community health extension workers and community volunteers

Training CHEWs and community volunteers before MDA ensures smooth implementation. There are three phases of training. The first two-day training session is for divisional coordinators and supervisors from all over the district that will carry out MDA. The second two-day training session is for CHEWs, who are team leaders at the distribution posts. Finally, there is a one-day training session for community volunteers.

Everyone is taught about trachoma and SAFE, the trachoma situation in the country and respective districts, the pharmacology of azithromycin (with emphasis on uses, doses and possible side effects), the mapping of the distribution area, dosing of azithromycin and the use of the height stick, writing patient details, and how to write the daily summary reports. The different duties of the divisional coordinators, supervisors, CHEWs and community volunteers are then clearly explained to each respective group.

## 5. Mass treatment

The CHEWs are responsible for all activities at the treatment posts. These include identifying the areas that have not been visited. In cases of side effects or adverse drug reactions, CHEWs give first aid and notify the Ministry of Health immediately.

During the MDA, a CHEW is paired with two community volunteers. One volunteer measures the height of each person who will receive treatment to establish the appropriate dose, and the second volunteer records the personal details and the dose the person will receive. The antibiotic (azithromycin) is administered by the CHEW according to height, and older people are also examined for trichiasis. At the end of each day the CHEW tabulates the number of people treated and the drugs used as well as any wastage, and sends the daily summary to the supervisor.

The supervisors and divisional coordinators have broadly similar roles, just at different levels. They work together to ensure every team has enough azithromycin to ensure smooth distribution and that unused drugs are returned to a central store. Supervisors collate the reports submitted by teams and send them to the coordinator they report to. Coordinators collate all the supervisor reports and send them to the DHMT.
